# Correction: Kingshott et al. Alteration of Metabolic Conditions Impacts the Regulation of IGF-II/H19 Imprinting Status in Prostate Cancer. *Cancers* 2021, *13*, 825

**DOI:** 10.3390/cancers18020204

**Published:** 2026-01-08

**Authors:** Georgina Kingshott, Kalina Biernacka, Alex Sewell, Paida Gwiti, Rachel Barker, Hanna Zielinska, Amanda Gilkes, Kathryn McCarthy, Richard M. Martin, J. Athene Lane, Lucy McGeagh, Anthony Koupparis, Edward Rowe, Jon Oxley, Jeff M. P. Holly, Claire M. Perks

**Affiliations:** 1 IGF & Metabolic Endocrinology Group, Translational Health Sciences, Bristol Medical School, Learning & Research Building, Southmead Hospital, Bristol BS10 5NB, UK; mdxkz@bristol.ac.uk (K.B.); mdrmh@bristol.ac.uk (R.B.); hanna.zielinska@hotmail.com (H.Z.); jeff.holly@bristol.ac.uk (J.M.P.H.); claire.m.perks@bristol.ac.uk (C.M.P.); 2 Department of Cellular Pathology, North Bristol NHS Trust, Southmead Hospital, Bristol BS10 5NB, UK; alexsewell@doctors.org.uk (A.S.); paida@doctors.org.uk (P.G.); jon.oxley@nbt.nhs.uk (J.O.); 3 Department of Pathology, North West Anglia NHS Foundation Trust, Peterborough PE3 9GZ, UK; 4 Department of Haematology, Cardiff University, Heath Park, Cardiff CF14 4XN, UK; gilkes@cardiff.ac.uk; 5 Department of Surgery, Department of Medicine, Southmead Hospital, Bristol BS10 5NB, UK; kathryn.mccarthy@nbt.nhs.uk; 6 Population Health Sciences, Bristol Medical School, University of Bristol, Canynge Hall, 39 Whatley Road, Bristol BS8 2PS, UK; richard.martin@bristol.ac.uk; 7 National Institute for Health Research, Biomedical Research Centre at University Hospitals Bristol and Weston NHS Foundation Trust and the University of Bristol, Biomedical Research Unit Offices, University Hospitals Bristol Education Centre, Dental Hospital, Lower Maudlin Street, Bristol BS1 2LY, UK; 8 Bristol Randomised Trials Collaboration, Population Health Sciences, Bristol Medical School, University of Bristol, Canynge Hall, 39 Whatley Road, Bristol BS8 2PS, UK; athene.lane@bristol.ac.uk; 9 Supportive Cancer Care Research Group, Faculty of Health and Life Sciences, Oxford Institute of Nursing, Midwifery and Allied Health Research, Oxford Brookes University, Jack Straws Lane, Marston, Oxford OX3 0FL, UK; lmcgeagh@brookes.ac.uk; 10 Department of Urology, Bristol Urological Institute, Southmead Hospital, Bristol BS10 5NB, UK; anthony.koupparis@nbt.nhs.uk (A.K.); edward.rowe@nbt.nhs.uk (E.R.)

## Error in Figure

In the original publication [1], there was a mistake in Figure 2A*ii*, which had inadvertently been partially duplicated using part of panel 1C. We have assembled a new Figure 2A*ii*. In addition, there was an overlap in Figure 5B between panels A (benign) and D (cancer). We have assembled a new representative Figure 5B to replace this panel, and the figure legend has been corrected. The authors state that the scientific conclusions are unaffected. This correction was approved by the Academic Editor. The original publication has also been updated.

## Supplementary Materials

The original uncropped Western Blot has been provided in the supplementary materials.

## Figures and Tables

**Figure 2:**
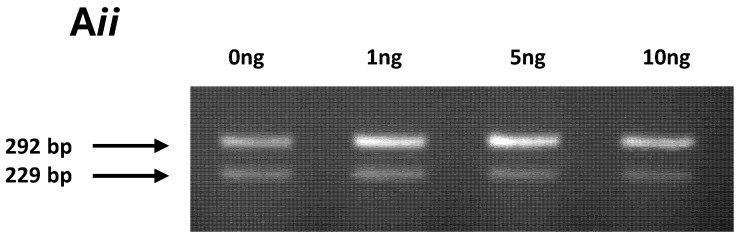
(**A*ii***) glucose and dosed with 0, 1, 5 and 10 ng/mL TNFα showed two bands sized 229 and 292 bp, indicating a change from mono- to biallelic IGF-II expression and, therefore, loss of imprinting. (*n* = 3).

**Figure 5:**
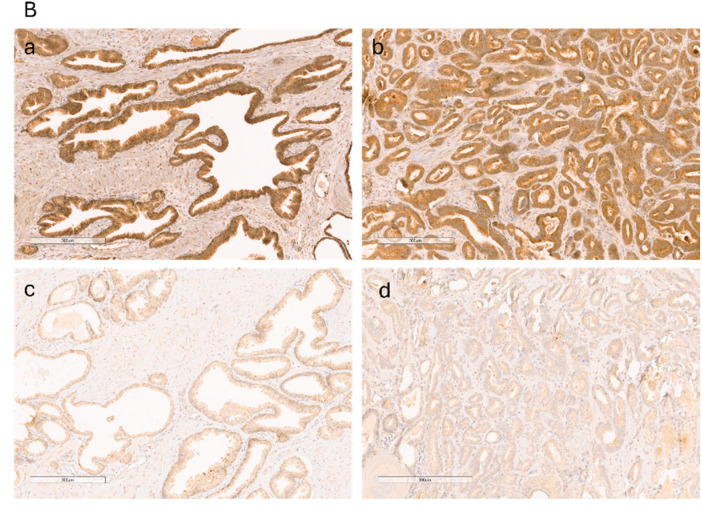
(**B**) Immunohistochemical analysis of IGF-II in two individuals, one with strong IGF-II staining in both benign (**a**) and cancer (**b**) tissue and one with weak IGF-II staining in both benign (**c**) and cancer (**d**) tissue. Scale bar = 300 um.

## References

[1] Kingshott G., Biernacka K., Sewell A., Gwiti P., Barker R., Zielinska H., Gilkes A., McCarthy K., Martin R.M., Lane J.A. (2021). Alteration of Metabolic Conditions Impacts the Regulation of IGF-II/H19 Imprinting Status in Prostate Cancer. Cancers.

