# Supplementary figures and images for: Alteration of Metabolic Conditions Impacts the Regulation of IGF-II/H19 Imprinting Status in Prostate Cancer
Source: Cancers (Basel). 2021 Feb 16;13(4):825. doi: 10.3390/cancers13040825 (PMC7920081; doi:10.3390/cancers13040825)

**Supplementary Materials Figure S1:**

original gel for fig 1C and 2A :

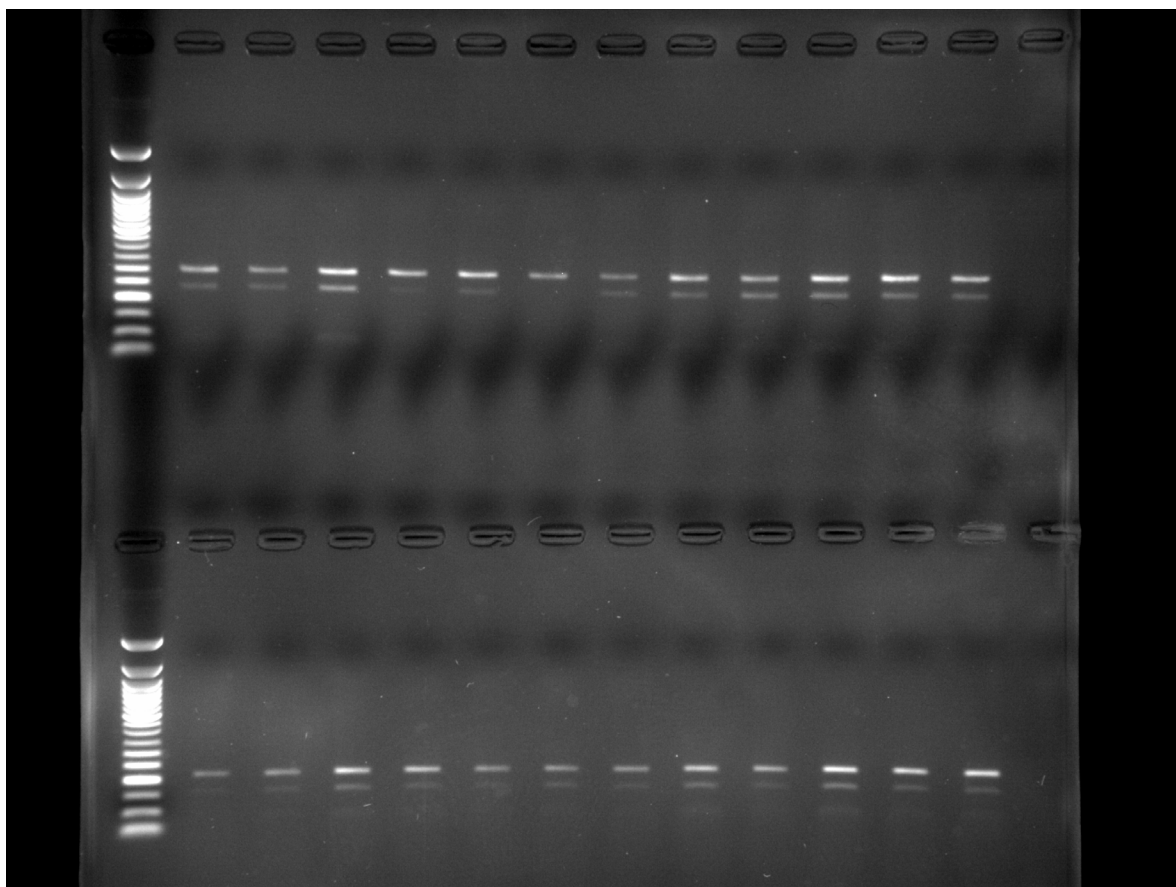

Supplement: Supplementary file 1 [file cancers-13-00825-s001.zip › cancers-1091219-Supplementary Materials.pdf]
